# Minimally Invasive Mitral Valve Surgery in Patients Aged ≥75 Years: An Expanding Standard of Care

**DOI:** 10.3390/jcm14165798

**Published:** 2025-08-16

**Authors:** Mariafrancesca Fiorentino, Elisa Mikus, Diego Sangiorgi, Simone Calvi, Antonino Costantino, Elena Tenti, Alberto Tripodi, Carlo Savini

**Affiliations:** 1 Cardiovascular Department, Maria Cecilia Hospital, GVM Care & Research, 48033 Cotignola, Italy; elisamikus@yahoo.it (E.M.); scalvi@gvmnet.it (S.C.); antonino@costantinorc.com (A.C.); etenti@gvmnet.it (E.T.); atripodi@gvmnet.it (A.T.); carlo.savini@unibo.it (C.S.); 2 Department of Experimental Diagnostic and Surgical Medicine (DIMEC), University of Bologna, 40126 Bologna, Italy

**Keywords:** minimally invasive surgery, mitral valve surgery, elderly patients

## Abstract

**Background:** Right anterior mini-thoracotomy has gained increasing popularity as a preferred approach for mitral valve surgery due to its numerous advantages. This study aims to evaluate the safety and efficacy of this technique in elderly patients. **Methods**: Between January 2010 and November 2024, a total of 4092 adult patients underwent mitral valve repair or replacement at our institution. Of these, 1687 patients were treated using a minimally invasive approach. This analysis focuses on elderly patients aged 75 years and older (*n* = 402), further subdivided into two groups: 75–79 years (*n* = 253) and 80 years and older (*n* = 149). **Results**: The study population comprised 49.8% male patients. A small percentage (1.7%) had a history of endocarditis, and 6.5% had undergone prior cardiac surgery. The median logistic EuroSCORE was 7.68 (IQR 5.83–11.00), and the median EuroSCORE II was 2.75 (1.71, 4.40). Alternative cannulation strategies, guided by AngioCT scans, can expand the applicability of this technique to patients unsuitable for femoral cannulation. Median durations for cardiopulmonary bypass (CPB) and aortic cross-clamping were 99.5 and 80.0 min, respectively. Median ventilation time was 7 h, and the median ICU stay was 2 days. Atrial fibrillation was the most common postoperative complication (20.9%). A significant proportion of patients (47.8%) required blood transfusions, and 3.0% needed re-exploration for bleeding. The in-hospital mortality rate was 3.7%, with 7 (1.7%) patients requiring postoperative dialysis and 5 (1.2%) experiencing sepsis and multiple organ failure. Patients aged 80 years and older exhibited worse renal function and higher EuroSCOREs compared to the younger group (*p* < 0.001). However, they had shorter CPB (*p* = 0.004) and cross-clamp times (*p* = 0.001) and underwent a higher proportion of valve replacements (*p* = 0.003). Rates of major complications and in-hospital mortality were comparable between the two age groups. Logistic regression analysis identified the logistic EuroSCORE as the only significant preoperative risk factor (*p* = 0.001). **Conclusions**: Right anterior minithoracotomy is a safe and reproducible surgical approach, even in elderly patients, promoting faster recovery with a lower risk of complications. Among patients aged >80 years, despite higher comorbidities and elevated EuroSCORE II, in-hospital outcomes are comparable to those aged 75–79 years.

## 1. Introduction

Since its introduction [1], the mini-thoracotomy approach for mitral valve surgery has gained widespread acceptance due to its consistently favorable impact on patient outcomes. Initially developed as an alternative to the conventional full sternotomy, this technique has demonstrated significant advantages in appropriately selected patients. As highlighted in a recent meta-analysis, although the approach is associated with longer cardiopulmonary bypass and cross-clamp times, it confers notable postoperative benefits. These include a lower incidence of renal failure, new onset atrial fibrillation, blood transfusion requirements, wound infections, and a shorter length of stay in both the intensive care unit (ICU) and overall hospitalization—factors that collectively contribute to reduced healthcare utilization and costs [2]. In addition to these clinical advantages, the minimally invasive technique is also associated with improved patient satisfaction. It is generally perceived to cause less postoperative pain, facilitate earlier mobilization and recovery, and result in superior cosmetic outcomes due to the avoidance of a midline sternotomy [3]. These factors are particularly relevant in the context of an aging surgical population that may be more vulnerable to postoperative complications and prolonged recovery times. For these reasons, although the mini-thoracotomy approach is not yet formally endorsed in current European or American guidelines on valvular heart disease management [4,5], it is increasingly regarded as the gold standard for mitral valve surgery across a broad spectrum of patients. In recent years, its indications have expanded beyond low-risk cohorts to include more complex and higher-risk subgroups. Notably, the technique is being increasingly adopted in patients with active infective endocarditis [6,7], those with elevated body mass index (BMI) [8,9], and in reoperative mitral valve procedures [10,11], particularly in patients with patent coronary artery bypass grafts, where re-entry via sternotomy may increase procedural risk due to potential graft injury. In parallel, the epidemiology of valvular heart disease (VHD) in developed countries has evolved dramatically over the past five decades. While rheumatic heart disease has become increasingly uncommon, the aging of the population has led to a marked rise in the prevalence of degenerative valve pathologies [12,13]. Consequently, the average age of patients referred for mitral valve intervention is steadily increasing, posing new challenges in terms of surgical risk, frailty, and recovery. Against this backdrop, the aim of the present study is to evaluate the feasibility, safety, and early outcomes of the minimally invasive mini-thoracotomy approach for mitral valve surgery in elderly patients—specifically focusing on those aged 75 years and older. Through this analysis, we seek to determine whether the benefits of this approach can be reliably extended to an aging population that may derive particular advantage from less invasive surgical strategies.

## 2. Materials and Methods

### 2.1. Study Population

All adult patients (age > 18 years) who underwent isolated or combined mitral valve surgery via a right-sided mini-thoracotomy at our institution were considered for inclusion in this study. No formal sample size calculation was performed; instead, all eligible patients meeting the criteria during the study period were included. The choice to perform surgery using a minimally invasive approach was left to the discretion of the operating surgeon. Between January 2010 and November 2024, a total of 4092 adult patients underwent mitral valve repair or replacement. Of these, 1687 procedures were performed using a minimally invasive approach. The present analysis focuses on a subset of elderly patients aged ≥75 years (*n* = 402), who were further stratified into two age groups: 75–79 years (*n* = 253) and ≥80 years (*n* = 149). The study was conducted in accordance with the principles of the Declaration of Helsinki and was approved by the Romagna Ethics Committee on 20 November 2019 (Protocol No. 9689/2019 I.5/186). Due to the retrospective nature of the study, the requirement for individual informed consent was waived. Clinical data were retrospectively collected from institutional medical records, including preoperative, intraoperative, and postoperative variables. Efforts were made to minimize missing data, and only complete cases were included in the final analysis. The primary endpoint was in-hospital mortality, defined as any death occurring prior to discharge from the index hospitalization. Secondary endpoints included the occurrence of any postoperative adverse events.

### 2.2. Operative Strategy

Surgical indications were established in accordance with the most recent guidelines of the American College of Cardiology/American Heart Association and the European Society of Cardiology/European Association for Cardio-Thoracic Surgery [4,5]. The surgical technique adopted in our institution has been previously described [9]. In brief, all procedures were performed under total intravenous anesthesia with selective lung ventilation via a double-lumen endotracheal tube. Transesophageal echocardiography (TEE) was systematically employed to assess cardiac and valvular function preoperatively and intraoperatively. Peripheral cannulation was achieved by exposing the femoral vessels through a 3 cm incision, with both the femoral artery and vein cannulated using the Seldinger technique under TEE guidance. In female patients, a 4 to 6 cm skin incision was made along the inframammary fold; in male patients, the incision was placed at the level of the fourth right intercostal space. A right anterior mini-thoracotomy was performed through the fourth intercostal space. Two accessory ports were inserted in the fourth and sixth intercostal spaces to accommodate the intracardiac suction, carbon dioxide insufflation, and thoracoscopic visualization. The pericardium was opened 2 to 3 cm anterior to the phrenic nerve, and traction sutures were applied to displace it toward the right hemithorax. Cardiopulmonary bypass (CPB) was initiated, and patients were cooled to a core temperature of 30–32 °C. Aortic cross-clamping was performed using either a Chitwood clamp introduced through a separate 10 mm incision or a Cygnet clamp positioned directly through the thoracotomy. Myocardial protection was achieved using antegrade cold blood cardioplegia (St. Thomas solution with procaine) or Custodiol solution delivered into the aortic root. In cases requiring tricuspid valve intervention, caval snares were applied around the venous cannulae prior to right atriotomy to ensure optimal exposure. Following cardioplegic arrest, the left atrium was accessed for mitral valve repair or replacement. If tricuspid valve surgery was indicated, it was performed after closure of the left atrium via a right atriotomy. Upon completion of the intracardiac procedure (s), temporary epicardial pacing wires were positioned on the right ventricle, the aorta was unclamped, and patients were weaned from CPB according to standard institutional protocols. Postoperative care mirrored the management protocols used for conventional full sternotomy procedures. Upon arrival in the intensive care unit (ICU), patients were assessed for extubation on postoperative day (POD) 0, based on chest tube drainage, neurological status, and gas exchange. If clinically stable, patients typically spent the first night in the ICU and were transferred to a medium care unit (MCU) on POD 1. Early mobilization to an armchair was initiated on POD 1, with chest drains generally removed on POD 2. Patients were then transferred to the general ward, where full ambulation was achieved. Discharge was typically feasible on POD 7, assuming an uneventful postoperative course.

### 2.3. Statistical Analysis

Continuous variables were assessed for normality using the Shapiro–Wilk test. As they did not follow a normal distribution, they were summarized as a median and interquartile range (Q1–Q3; 1st–3rd quartile) and compared using the Kruskal–Wallis test. Categorical variables were presented as absolute counts and percentages and analyzed with Fisher’s exact test. To identify factors associated with mortality, LASSO (Least Absolute Shrinkage and Selection Operator) regression with 300-fold cross-validation was used. Model performance was assessed by calculating the area under the receiver operating characteristic curve (AUC). All analyses were conducted using R 4.4.0 (R Foundation for Statistical Computing, Vienna, Austria), with a significance threshold of *p* < 0.05.

## 3. Results

### 3.1. Patients’ Characteristics

Patients’ characteristics are presented in Table 1. The median age of our population was 72 years, and 202 patients (50.2%) were female. Obviously, many patients presented with the most common cardiovascular risk factors, including hypertension (86.1%), diabetes (50%), dyslipidemia (63.9%), and active or previous smoking (50%). The majority of patients were in good preoperative clinical conditions, with a median ejection fraction of 59%, a median creatinine level of 1.01 mg/dL, and a median EuroSCORE II of 2.80. Four patients (11.1%) had a history of preoperative stroke, and two (5.6%) had experienced a transient ischemic attack (TIA). One patient (2.8%) underwent a redo operation, and another (2.8%) was supported by an intra-aortic balloon pump prior to surgery. The etiology of mitral regurgitation in our patient population was predominantly organic, but the cohort includes all patients who underwent mitral valve surgery, including those with functional mitral regurgitation of either ventricular or atrial origin. Following established guidelines, patients with functional mitral regurgitation at our center were first treated with optimal medical therapy, including ventricular resynchronization for the ventricular functional form and aggressive control of atrial fibrillation for cases of atrial etiology. Only after medical therapy has been fully optimized, and if severe regurgitation persists with criteria for surgery, are patients considered for an operation. Preoperative patient characteristics stratified by age group are summarized in Table 1. The overall median age was 78 years (interquartile range [IQR], 76–82). Among the 402 patients included in the study, 253 (62.9%) were aged between 75 and 79 years, while 149 patients (37.1%) were 80 years or older. The cohort comprised 49.8% male patients. A prior history of infective endocarditis was present in 1.7% of the population, and 6.5% had previously undergone cardiac surgery. The median logistic EuroSCORE was 7.68 (IQR 5.83–11.00), and the median EuroSCORE II was 2.75 (IQR 1.71–4.40). Sex distribution was balanced between the two age groups, with no significant differences observed. Similarly, the prevalence of major cardiovascular risk factors—such as hypertension, dyslipidemia, and diabetes mellitus—as well as smoking history and chronic obstructive pulmonary disease (COPD) was comparable between groups. Functional status was also similar: the majority of patients in both groups were classified as New York Heart Association (NYHA) class II or III at the time of surgery. The median left ventricular ejection fraction (LVEF) across the cohort was 60% (IQR 55–65), with no relevant intergroup differences. The incidence of active infective endocarditis, history of stroke, and prior cardiac surgery did not differ significantly between age groups (*p* = 0.713, *p* = 0.716, and *p* = 0.207, respectively). However, patients aged ≥80 years had significantly worse preoperative renal function (*p* < 0.001) and higher surgical risk scores, including both the logistic EuroSCORE and EuroSCORE II (*p* < 0.001), suggesting a frailer clinical profile in this subgroup.

### 3.2. In-Hospital Outcomes

Intraoperative and postoperative outcomes are detailed in Table 2. In the whole population, the median cardiopulmonary bypass (CPB) time was 99.5 min, and the median aortic cross-clamp time was 80.0 min. The percentage of mitral valves that underwent repair in the whole population was 290 (72.1%), with a significantly higher incidence of repair (*p* = 0.003) in the younger population (75–79 years) (77.5%) compared to those over 80 years old (63.1%). Combined procedures were performed in 75 (18.7%) patients in the whole population, with no significant differences across the age groups. Specifically, the combined procedures primarily included left atrial appendage closure, patent foramen ovale (PFO) closure, or tricuspid valve repair. We also performed a limited number of combined mitral and aortic valve surgeries using a minimally invasive approach, as detailed in our previous publication [14]. Patients had a median ventilation time of 7 h and a median ICU stay of 2 days. Atrial fibrillation was the most frequent postoperative complication, occurring in 20.9% of patients. Blood transfusion was required in 47.8% of cases, while 3.0% of patients underwent surgical re-exploration due to bleeding. The overall in-hospital mortality rate was 3.7%. Postoperative dialysis was required in 7 patients (1.7%), and 5 patients (1.2%) experienced sepsis with subsequent multi-organ failure. Interestingly, patients aged ≥80 years had significantly shorter CPB (*p* = 0.004) and cross-clamp times (*p* = 0.001) compared to the younger cohort, likely due to a higher proportion of valve replacements rather than repairs performed in this subgroup (*p* = 0.003).

Despite their higher preoperative risk profile, elderly patients experienced comparable rates of major postoperative complications and in-hospital mortality relative to their younger counterparts. The only exception was a longer ICU stay observed in the ≥80 group (*p* < 0.05), although this did not translate into prolonged overall hospitalization. Multivariable logistic regression analysis for predictors of in-hospital mortality (Table 3) identified the logistic EuroSCORE as the only significant independent preoperative risk factor (*p* = 0.001).

To assess the impact of our center’s growing surgical experience on patient outcomes, we compared two distinct time periods: 2010–2017 and 2018–2024. The detailed results are presented in dedicated tables within the Supplemental Materials (Tables S1 and S2). Preoperative clinical characteristics were largely comparable between the two groups, with the exception of a higher proportion of male patients (53.7% vs. 42.8% *p* = 0.038) and a greater prevalence of systemic arterial hypertension (80.9% vs. 65.5% *p* = 0.001) in the latter period. Regarding surgical outcomes, we observed an increase over time in both cardiopulmonary bypass (116 vs. 74 min) and aortic cross-clamp (88 vs. 62 min) durations (*p* < 0.001), and there was an increased proportion of valve repairs (75.5% vs. 66.2%) and a reduction in mitral valve replacements (24.5% vs. 33.8%) (*p* = 0.05). Despite these technical shifts, postoperative results improved, with the exception of a significant reduction in the incidence of blood cell transfusions (*p* = 0.005), postoperative atrial fibrillation (*p* = 0.015), a shorter median ventilation time (*p* < 0.001), and a lower proportion of patients requiring prolonged ventilation (*p* = 0.032).

## 4. Discussion

In this study, we present our fourteen-year experience with the minimally invasive approach to mitral valve surgery, with a specific focus on elderly patients. Over time, growing surgical expertise and advancements in technology have facilitated the extension of this approach to broader patient populations [2], including individuals traditionally considered high-risk, such as older adults. In this subset, reducing surgical trauma and minimizing the physiological burden of the intervention may translate into improved outcomes. Moreover, the minimally invasive approach for mitral valve disease is widely used, even in other patient categories considered fragile, such as obese patients and patients with endocarditis, as we have previously published [7,8,9]. However, this approach is not yet commonly used in an acute setting, such as for acute mitral regurgitation caused by myocardial infarction. In these cases, the prevailing surgical treatment is still the conventional approach via median sternotomy. This is due to the fact that these patients are often hemodynamically unstable, supported by mechanical devices (primarily IABP), and present with pulmonary edema that makes the single-lung ventilation required for a minimally invasive approach impractical. Furthermore, patients with acute ischemic mitral regurgitation often require combined treatment for coronary artery disease via coronary artery bypass grafting, which necessitates the conventional surgical approach. Promising results in this specific context seem to be emerging from the use of a transcatheter approach (TEER), as reported in a recent meta-analysis [15]. The potential value of a minimally invasive strategy in elderly patients is further underscored by the physiological changes associated with aging. Aging of the cardiovascular system is characterized by progressive structural and functional alterations, including increased arterial stiffness, myocardial fibrosis, impaired diastolic function, and endothelial dysfunction. These changes contribute to elevated left ventricular afterload, reduced coronary flow reserve, and decreased cardiac adaptability to stress. Consequently, elderly patients typically exhibit a reduced physiological reserve, making them more vulnerable to perioperative complications and highlighting the potential advantage of less invasive surgical techniques [16]. The application of minimally invasive mitral valve surgery in elderly patients has been previously addressed in the literature, with studies comparing the mini-thoracotomy approach to conventional full sternotomy or evaluating outcomes across different age groups. However, some aspects remain to be clarified. First, there is no standardized definition of “elderly” in the context of cardiac surgery. While some studies use 65 years as a threshold [17,18], others focus specifically on octogenarians [19,20]. Given current demographic trends and the age distribution of our cohort, we propose that patients aged ≥75 years be considered representative of the elderly population in this context [21]. Furthermore, it is well established that operative mortality and morbidity increase with advancing age in patients undergoing mitral valve replacement [22]. While this is partly attributable to a greater burden of comorbidities and procedural complexity, age itself remains an independent predictor of operative risk. These considerations reinforce the relevance of exploring strategies—such as minimally invasive approaches—that may mitigate surgical stress and optimize outcomes in this increasingly prevalent patient group. With regard to studies comparing the minimally invasive approach to conventional median sternotomy for mitral valve surgery in elderly patients, a recently published meta-analysis [17] demonstrated that patients treated via a minimally invasive approach had significantly lower odds of acute renal failure, prolonged mechanical ventilation, and the need for blood transfusion. They also experienced shorter lengths of stay both in the intensive care unit and overall hospitalization, with no significant differences in the rates of in-hospital mortality, stroke, respiratory infections, reoperation for bleeding, or postoperative atrial fibrillation between the two approaches. It is worth noting that the minimally invasive technique was associated with longer cardiopulmonary bypass and aortic cross-clamp times. However, this apparent drawback did not translate into worse clinical outcomes, suggesting that, even in elderly patients, the physiological impact of prolonged operative times may be offset by the reduced surgical trauma inherent to the mini-thoracotomy approach. Similar findings were reported by Iribarne et al. [21], who compared minimally invasive and conventional mitral valve surgery and confirmed that the minimally invasive approach was associated with longer cross-clamp and cardiopulmonary bypass time. However, patients in the minimally invasive group experienced a significantly shorter hospital stay, with no significant differences in major postoperative complications or long-term survival. This approach was also linked to a lower median hospitalization cost and a higher likelihood of being discharged home rather than to a rehabilitation facility with patients achieving faster recovery in terms of independent ambulation and sit-to-stand transitions. At our institution, we are strong proponents of the minimally invasive approach for mitral valve surgery, believing its benefits are most pronounced in high-risk patient populations, such as the elderly. As such, all mitral valve procedures at our center are performed via a minimally invasive approach, unless specific contraindications are present. In line with this philosophy, we deliberately chose not to compare patients undergoing minimally invasive surgery with those treated via median sternotomy, as these two populations would have been inherently different and not readily comparable. A comparable investigation was recently published by Francica et al. [18], who compared outcomes between younger (<65 years) and older (>75 years) patients undergoing minimally invasive mitral valve surgery. As expected, elderly patients presented with a greater burden of comorbidities and a significantly higher EuroSCORE II. While postoperative mortality did not differ significantly between groups, elderly patients experienced a higher incidence of complications such as re-exploration for bleeding, stroke, reintubation, and the need for hemodialysis or blood transfusion. However, after propensity score matching, no significant differences in any postoperative outcomes were observed. These findings underscore that, when appropriately selected, elderly patients can derive similar clinical benefit from the minimally invasive approach without compromising safety or outcomes. Regarding patients aged over 80 years, it is well established that elective mitral valve repair can be performed with low operative mortality and favorable long-term outcomes in carefully selected octogenarians with degenerative mitral disease. Furthermore, mitral repair has been associated with superior long-term survival compared to valve replacement in this age group [19]. The minimally invasive approach has also been investigated within this subset of patients [20], and survival analysis identified age ≥ 84 years and serum creatinine ≥1.22 mg/d as thresholds associated with poorer prognosis, while female sex and a history of hypertension were independent predictors of the composite outcome. The authors concluded that age alone should not be regarded as a contraindication to minimally invasive mitral surgery. Instead, careful preoperative assessment aimed at identifying patients most likely to derive survival and functional benefit from surgery is essential to optimize outcomes and avoid futile interventions. In our study, we specifically analyzed a cohort of patients aged over 75 years who underwent minimally invasive mitral valve surgery and observed favorable in-hospital outcomes. The median ventilation time was 7 h, and the median ICU stay was 2 days. Atrial fibrillation was the most frequent postoperative complication (20.9%), blood transfusions were required in 47.8% of cases, and 3.0% of patients underwent surgical re-exploration for bleeding. The overall in-hospital mortality rate was 3.7%. Additionally, postoperative dialysis was required in 1.7% of patients, and 1.2% developed sepsis with subsequent multi-organ failure. We further stratified our cohort into two age groups (75–79 years vs. ≥80 years) to better understand age-related differences in outcomes. As expected, patients aged ≥80 years had significantly worse preoperative renal function and higher surgical risk profiles, including both the logistic EuroSCORE and EuroSCORE II. Despite these differences, there were no statistically significant differences in the incidence of major postoperative complications or in-hospital mortality between the two groups. The only notable difference was a longer ICU stay observed in patients aged ≥80 years, which can likely be attributed to the need for more intensive monitoring in this older, higher-risk subgroup. However, this did not translate into a prolonged overall hospital length of stay. Interestingly, patients aged ≥80 years had significantly shorter cardiopulmonary bypass (*p* = 0.004) and aortic cross-clamp times (*p* = 0.001) compared to the younger cohort. This finding is likely attributable to a higher proportion of valve replacements rather than repairs performed in the older subgroup (*p* = 0.003), which may partially explain the absence of increased mortality in the setting of advanced age and elevated surgical risk. To assess the impact of our center’s growing surgical experience on patient outcomes, we also compared two time periods (2010–2017 vs. 2018–2024). Preoperative characteristics were comparable, except for a higher proportion of male patients and a greater prevalence of systemic hypertension in the second period. Regarding outcomes, we observed an increase over time in cardiopulmonary bypass and aortic cross-clamp durations (*p* < 0.001). While this may initially appear counterintuitive in light of the expected surgical learning curve, the difference is likely attributable to an increased proportion of valve repairs versus replacements, and, more importantly, to changes in surgical techniques. In the earlier period, procedures were predominantly performed under direct vision via right thoracotomy, whereas the later period saw a greater adoption of a totally endoscopic approach. Furthermore, while the minimally invasive approach was initially limited to a small number of highly experienced surgeons, in the latter period this technique was increasingly adopted by the entire surgical team. Despite these changes, postoperative outcomes improved, with a reduced incidence of RBC transfusions, postoperative atrial fibrillation, a shorter median ventilation time, and fewer cases requiring prolonged ventilation. While our findings are consistent with previously published studies, we believe our data is particularly significant for two main reasons. First, they demonstrate in a large patient population that minimally invasive mitral valve surgery is both effective and safe for older patients. Secondly, we specifically show this to be true for patients both over 75 and over 80 years of age. However, in this subset of patients, preoperative screening is of paramount importance and must include computed tomography angiography (CTA) of the thoracoabdominal aorta [23], which can detect vascular pathologies otherwise undetectable during routine preoperative evaluation [24]. Such pathologies may increase the risk of perioperative stroke due to retrograde perfusion from femoral arterial cannulation. Nevertheless, a contraindication to femoral cannulation does not preclude the use of a minimally invasive approach, which can be performed using antegrade perfusion via the axillary artery [25]. This technique is increasingly adopted as the indication for minimally invasive surgery expand, particularly among the elderly population with a higher prevalence of atherosclerosis.

### Limitations

The main limitation of this study is its retrospective nature. All data represent a single-center experience, and the decision to use a minimally invasive approach was left to the surgeon’s discretion. Furthermore, as explained in the discussion section, the patients were not compared to those undergoing the same surgery through conventional sternotomy. Additionally, we do not have the absence of long-term follow-up data that would have further strengthened this study. Future studies should aim to assess survival, valve-related outcomes, and quality of life over time to further support this approach in elderly populations.

## 5. Conclusions

In our single-center experience, a minimally invasive approach through a right-sided mini-thoracotomy has proven to be both a safe and feasible approach for mitral valve surgery, even in elderly patients, promoting faster recovery with a lower risk of complications. Moreover, among patients aged ≥80 years, despite higher comorbidities and elevated EuroSCORE II, in-hospital outcomes are comparable to those aged 75–79 years. Nevertheless, the success of minimally invasive mitral valve surgery in elderly patients critically depends on meticulous patient selection. Comprehensive preoperative screening, including detailed vascular imaging and risk stratification, is mandatory to tailor the surgical strategy and minimize perioperative risks. This personalized approach is essential to optimize clinical outcomes and expand the indications of minimally invasive techniques safely across diverse patient populations. Further multicenter studies with larger cohorts and longer follow-up are warranted to validate these findings and refine patient selection criteria.

## Figures and Tables

**Table 1:** Preoperative characteristics.

N	>75 (402)	75–79 (253)	≥80 (149)	*p*
**Age median (** **IQR)**	**78 (76–81)**	**77 (76–78)**	**82 (81–83)**	**<0.001**
Male sex	200 (49.8)	127 (50.2)	73 (49)	0.837
Weight median (IQR)	68.00 [60.00, 77.00]	70.00 [60.00, 78.00]	65.00 [58.00, 75.00]	0.057
Hypertension	303 (75.4)	187 (73.9)	116 (77.9)	0.403
Diabetes	47 (11.7)	31 (12.3)	16 (10.7)	0.749
Dyslipidemia	204 (50.7)	129 (51.0)	75 (50.3)	0.918
Smoking	108 (26.9)	73 (28.9)	35 (23.5)	0.294
COPD	29 (7.2)	16 (6.3)	13 (8.7)	0.426
Preoperative pacemaker	6 (1.5)	3 (1.2)	3 (2.0)	0.674
Left ventricle Ejection Fraction median (IQR)	60.00 [55.00, 65.00]	60.00 [55.00, 65.00]	60.00 [55.00, 65.00]	0.797
Active endocarditis	7 (1.7)	4 (1.6)	3 (2.0)	0.713
Previous Stroke	8 (2.0)	6 (2.4)	2 (1.3)	0.716
Previous TIA	4 (1.0)	2 (0.8)	2 (1.3)	0.629
**Plasma creatinine (mg/dl) median (** **IQR)**	**1.00 [0.87, 1.24]**	**0.99 [0.84, 1.18]**	**1.08 [0.92, 1.27]**	**0.001**
Previous cardiac surgery	26 (6.5)	13 (5.1)	13 (8.7)	0.207
**Euroscore Logistic (%) median (** **IQR)**	**7.68 [5.83, 11.00]**	**6.59 [5.14, 9.90]**	**8.97 [7.84, 13.09]**	**<0.001**
**Euroscore II (%) median (** **IQR)**	**2.75 [1.71, 4.40]**	**2.19 [1.50, 3.65]**	**3.57 [2.39, 5.38]**	**<0.001**

IQR: Interquartile Range; COPD: Chronic Obstructive Pulmonary Disease; TIA: Transient Ischemic Attack. BOLD was used to emphasize statistically significant differences

**Table 2:** In-hospital outcomes.

N	>75–79 (402)	75–79 (253)	≥80 (149)	*p*
**Mitral valve replacement**	**112 (27.9)**	**57 (22.5)**	**55 (36.9)**	**0.003**
**Mitral valve repair**	**290 (72.1)**	**196 (77.5)**	**94 (63.1)**	**0.003**
Combined surgery	75 (18.7)	51 (20.2)	24 (16.1)	0.355
**CPB time median** **(** **IQR)**	**99.50 [77.00, 129.00]**	**104.00 [79.25, 135.00]**	**93.00 [71.75, 118.75]**	**0.004**
**Aortic cross-clamp time median** **(** **IQR)**	**80.00 [62.00, 105.00]**	**85.00 [66.00, 109.00]**	**74.00 [59.00, 88.25]**	**0.001**
In-hospital mortality	15 (3.7)	7 (2.8)	8 (5.4)	0.275
Postoperative stroke	6 (1.5)	3 (1.2)	3 (2.0)	0.674
Peri operative myocardial infarction	1 (0.2)	1 (0.4)	0 (0.0)	1.000
Postoperative inotropic support	43 (10.7)	24 (9.5)	19 (12.8)	0.320
New onset atrial fibrillation	84 (20.9)	52 (20.6)	32 (21.5)	0.899
Permanent pacemaker implantation	9 (2.2)	4 (1.6)	5 (3.4)	0.301
RBC transfusions	192 (47.8)	119 (47.0)	73 (49.0)	0.757
Re-thoracotomy for bleeding	12 (3.0)	6 (2.4)	6 (4.0)	0.373
Chest drain output in 24/hours median (IQR)	400.00 [262.50, 600.00]	425.00 [300.00, 600.00]	400.00 [250.00, 550.00]	0.739
Dialysis	7 (1.7)	3 (1.2)	4 (2.7)	0.431
Ventilation time median (IQR)	7.00 [5.00, 12.00]	8.00 [5.00, 11.25]	7.00 [5.00, 13.00]	0.515
Prolonged mechanical ventilation	53 (13.2)	31 (12.3)	22 (14.8)	0.542
Sepsis	5 (1.2)	3 (1.2)	2 (1.3)	1.000
**ICU length of stay median** **(** **IQR)**	**2.00 [2.00, 3.00]**	**2.00 [2.00, 3.00]**	**2.00 [2.00, 4.00]**	**0.045**
In-hospital length of stay median (IQR)	8.00 [7.00, 10.00]	8.00 [7.00, 10.00]	8.00 [7.00, 11.00]	0.935

IQR: Interquartile Range; CPB: Cardiopulmonary Bypass; RBC: Red Blood Cells; ICU: Intensive Care Unit.

**Table 3:** Multivariable logistic regression analysis.

In-Hospital Mortality	OR	95% CI		*p*
Active endocarditis	4.1	0.6	30.1	0.162
**Euroscore log**	**1.1**	**1.0**	**1.1**	**0.013**

## Data Availability

The data presented in this study is available on request from the corresponding author. The data is not publicly available due to data protection directive 95/46/EC.

## Supplementary Materials

The following supporting information can be downloaded at: https://www.mdpi.com/article/10.3390/jcm14165798/s1, Table S1: Preoperative characteristics according to time period; Table S2: Intra and postoperative characteristics according to time period.

## References

[1] Casselman F.P., Van Slycke S., Dom H., Lambrechts D.L., Vermeulen Y., Vanermen H. (2003). Endoscopic mitral valve repair: Feasible, reproducible, and durable. J. Thorac. Cardiovasc. Surg..

[2] Al Shamry A., Jegaden M., Ashafy S., Eker A., Jegaden O. (2023). Minithoracotomy versus sternotomy in mitral valve surgery: Meta-analysis from recent matched and randomized studies. J. Cardiothorac. Surg..

[3] Karangelis D., Loggos S., Mitropoulos F. (2021). Minimally Invasive versus Conventional Mitral Valve Surgery. A Clinical Equipoise or Not Really?. J. Investig. Surg..

[4] Vahanian A., Beyersdorf F., Praz F., Milojevic M., Baldus S., Bauersachs J., Capodanno D., Conradi L., De Bonis M., De Paulis R. (2022). ESC/EACTS Scientific Document Group. 2021 ESC/EACTS Guidelines for the management of valvular heart disease. Eur. Heart J..

[5] Otto C.M., Nishimura R.A., Bonow R.O., Carabello B.A., Erwin J.P., Gentile F., Jneid H., Krieger E.V., Mack M., McLeod C. (2021). 2020 ACC/AHA Guideline for the Management of Patients with Valvular Heart Disease: Executive Summary: A Report of the American College of Cardiology/American Heart Association Joint Committee on Clinical Practice Guidelines. Circulation.

[6] Raikar C., Wolfe S., Lagazzi L.F., Darehzereshki A., Kister N., Wei L., Badhwar V., Mehaffey J.H. (2025). Minimally Invasive Valve Surgery for Patients With Infective Endocarditis: A Comparative Study. Ann. Thorac. Surg..

[7] Mikus E., Fiorentino M., Sangiorgi D., Costantino A., Calvi S., Tenti E., Tremoli E., Tripodi A., Savini C. (2025). Minimally Invasive Versus Full Sternotomy Approaches in Mitral Valve Surgery for Infective Endocarditis: A Retrospective Comparative Analysis. Diseases.

[8] Ali-Hasan-Al-Saegh S., Helms F., Aburahma K., Takemoto S., De Manna N.D., Amanov L., Ius F., Karsten J., Zubarevich A., Schmack B. (2024). Can Obesity Serve as a Barrier to Minimally Invasive Mitral Valve Surgery? Overcoming the Limitations-A Multivariate Logistic Regression Analysis. J. Clin. Med..

[9] Fiorentino M., Mikus E., Sangiorgi D., Tripodi A., Calvi S., Tenti E., Costantino A., Savini C. (2025). Does Body Mass Index Impact Outcomes in Patients Undergoing Minimally Invasive Mitral Valve Surgery?. Medicina.

[10] Tariq M.A., Malik M.K., Uddin Q.S., Altaf Z., Zafar M. (2023). Minimally Invasive Procedure versus Conventional Redo Sternotomy for Mitral Valve Surgery in Patients with Previous Cardiac Surgery: A Systematic Review and Meta-Analysis. J. Chest Surg..

[11] Barbero C., Pocar M., Costamagna A., Capozza C., Aloi V., Stura E.C., Salizzoni S., Rinaldi M. (2024). Endo-Aortic Clamp for Minimally Invasive Redo Mitral Valve Surgery: Early Outcome. J. Cardiovasc. Dev. Dis..

[12] d’ARcy J.L., Prendergast B.D., Chambers J.B., Ray S.G., Bridgewater B. (2011). Valvular heart disease: The next cardiac epidemic. Heart.

[13] Aluru J.S., Barsouk A., Saginala K., Rawla P., Barsouk A. (2022). Heart Disease Epidemiology. Med. Sci..

[14] Fiorentino M., Mikus E., Tripodi A., Sangiorgi D., Calvi S., Tenti E., Costantino A., Savini C. (2025). Combined Mitral and Aortic Valve Surgery Through a Right Minithoracotomy: A Single-Center Experience. Innovations.

[15] Pyrpyris N., Dimitriadis K., Theofilis P., Iliakis P., Beneki E., Pitsiori D., Tsioufis P., Shuvy M., Aznaouridis K., Tsioufis K. (2024). Transcatheter Structural Heart Interventions in the Acute Setting: An Emerging Indication. J. Clin. Med..

[16] Yaffee D.W., Williams M.R. (2016). Cardiovascular Surgery in the Elderly. Semin. Thorac. Cardiovasc. Surg..

[17] Hage A., Hage F., Al-Amodi H., Gupta S., Papatheodorou S.I., Hawkins R., Ailawadi G., Mittleman M.A., Chu M.W.A. (2021). Minimally Invasive Versus Sternotomy for Mitral Surgery in the Elderly: A Systematic Review and Meta-Analysis. Innov. Technol. Tech. Cardiothorac. Vasc. Surg..

[18] Francica A., Barbero C., Tonelli F., Cerillo A.G., Lodo V., Centofanti P., Marchetto G., Di Credico G., De Paulis R., Stefano P. (2024). Minimally Invasive Mitral Valve Surgery in Elderly Patients: Results from a Multicenter Study. J. Clin. Med..

[19] Chikwe J., Goldstone A.B., Passage J., Anyanwu A.C., Seeburger J., Castillo J.G., Filsoufi F., Mohr F.W., Adams D.H. (2011). A propensity score-adjusted retrospective comparison of early and mid-term results of mitral valve repair versus replacement in octogenarians. Eur. Hear. J..

[20] Barbero C., Brenna D., Salsano A., Pocar M., Stura E.C., Calia C., Sebastiano V., Rinaldi M., Ricci D. (2023). Minimally invasive valve surgery: Pushing boundaries over the eighty. J. Geriatr. Cardiol..

[21] Iribarne A., Easterwood R., Russo M.J., Chan E.Y., Smith C.R., Argenziano M. (2012). Comparative effectiveness of minimally invasive versus traditional sternotomy mitral valve surgery in elderly patients. J. Thorac. Cardiovasc. Surg..

[22] Mehta R.H., Eagle K.A., Coombs L.P., Peterson E.D., Edwards F.H., Pagani F.D., Deeb G., Bolling S.F., Prager R.L. (2002). Influence of age on outcomes in patients undergoing mitral valve replacement. Ann. Thorac. Surg..

[23] Cocchieri R., Mousavi I., Verbeek E.C., Riezebos R.K., Yazdanbakhsh A.P., Mol B.A.M.J.d. (2024). Elderly patients benefit from minimally invasive mitral valve surgery: Perioperative risk management matters. Interdiscip. Cardiovasc. Thorac. Surg..

[24] Immohr M.B., Sugimura Y., Kröpil P., Aubin H., Minol J.-P., Albert A., Boeken U., Lichtenberg A., Akhyari P. (2021). Impact of standardized computed tomographic angiography for minimally invasive mitral and tricuspid valve surgery. J. Cardiothorac. Surg..

[25] Petersen J., Naito S., Kloth B., Pecha S., Zipfel S., Alassar Y., Detter C., Conradi L., Reichenspurner H., Girdauskas E. (2022). Antegrade axillary arterial perfusion in 3D endoscopic minimally-invasive mitral valve surgery. Front. Cardiovasc. Med..

