# Supplementary material for: Minimally Invasive Mitral Valve Surgery in Patients Aged ≥75 Years: An Expanding Standard of Care
Source: J Clin Med. 2025 Aug 16;14(16):5798. doi: 10.3390/jcm14165798 (PMC12387068; doi:10.3390/jcm14165798)
Supplement: Supplementary file 1 [file jcm-14-05798-s001.zip › jcm-3788052-supplementary.pdf]

Table S1: Preoperative characteristics according to time period

	2010-2017	2018-2024	p
n	145	257	
Age median (IQR)	79.00 [76.00, 81.00]	78.00 [76.00, 81.00]	0.240
**Male sex**	**62 (42.8)**	**138 (53.7)**	**0.038**
Weight median (IQR)	66.00 [60.00, 76.00]	70.00 [60.00, 79.00]	0.202
**Hypertension**	**95 (65.5)**	**208 (80.9)**	**0.001**
Diabetes	13 (9.0)	34 (13.2)	0.258
Dyslipidemia	71 (49.0)	133 (51.8)	0.605
Smoking	34 (23.4)	74 (28.8)	0.292
COPD	7 (4.8)	22 (8.6)	0.228
Preoperative pacemaker	2 (1.4)	4 (1.6)	1.000
Left ventricle Ejection Fraction median (IQR)	60.00 [55.00, 65.00]	60.00 [55.00, 65.00]	0.791
Active endocarditis	2 (1.4)	5 (1.9)	1.000
Previous Stroke	5 (3.4)	3 (1.2)	0.143
Previous TIA	3 (2.1)	1 (0.4)	0.136
Plasma creatinine (mg/dl) median (IQR)	1.00 [0.88, 1.31]	1.00 [0.87, 1.19]	0.197
Previous cardiac surgery	5 (3.4)	21 (8.2)	0.090
Euroscore Logistic (%) median (IQR)	7.16 [6.19, 9.80]	7.94 [5.82, 11.10]	0.870
Euroscore II (%) median (IQR)	2.94 [1.86, 4.97]	2.68 [1.63, 4.21]	0.198

Table S2: Intra and postoperative characteristics according to time period

	2010-2017	2018-2024	p
n	145	257	
**Mitral valve replacement**	**49 (33.8)**	**63 (24.5)**	**0.050**
**Mitral valve repair**	**96 (66.2)**	**194 (75.5)**	**0.050**
Combined surgery	32 (22.1)	43 (16.7)	0.230
**CPB time median (IQR)**	**74.00 [59.00, 100.00]**	**116.00 [91.00, 143.50]**	**<0.001**
**Aortic cross-clamp time median (IQR)**	**62.00 [47.50, 82.50]**	**88.00 [71.25, 111.75]**	**<0.001**
In-hospital mortality	8 (5.5)	7 (2.7)	0.176
Postoperative stroke	4 (2.8)	2 (0.8)	0.194
Peri operative myocardial infarction	0 (0.0)	1 (0.4)	1.000
Postoperative inotropic support	18 (12.4)	25 (9.7)	0.406
**New onset atrial fibrillation**	**40 (27.6)**	**44 (17.1)**	**0.015**
Permanent pacemaker implantation	3 (2.1)	6 (2.3)	1.000
**RBC transfusions**	**83 (57.2)**	**109 (42.4)**	**0.005**
Re-thoracotomy for bleeding	5 (3.4)	7 (2.7)	0.763
Chest drain output in 24/hours median (IQR)	400.00 [300.00, 650.00]	400.00 [250.00, 600.00]	0.142
Dialysis	4 (2.8)	3 (1.2)	0.258
**Ventilation time median (IQR)**	**9.00 [6.00, 16.00]**	**6.00 [5.00, 10.00]**	**<0.001**
**Prolonged mechanical ventilation**	**12 (8.3)**	**41 (16.0)**	**0.032**
Sepsis	0 (0.0)	5 (1.9)	0.164
ICU length of stay median (IQR)	2.00 [1.83, 3.64]	2.00 [2.00, 3.00]	0.475
In-hospital length of stay median (IQR)	8.00 [7.00, 11.00]	8.00 [7.00, 10.00]	0.382
